# Feasibility of conditional urine culture based on pathological urinalysis: a retrospective diagnostic stewardship study

**DOI:** 10.1017/ash.2026.10906

**Published:** 2026-10-16

**Authors:** Regev Cohen, Shelly Lipman-Arens, Yael Galnoor-Tene, Linor Ishay, Olga Feld-Simon, Orna Ben-Natan, Aliza Vaknin, Mohammed Ganayem, Yana Tal, Limor Rabinovitch, Milena Pitashny, Elias Tannous, Maanit Shapira

**Affiliations:** 1 The Ruth and Bruce Rappaport Faculty of Medicine, Technion Israel Institute of Technology, Haifa, Israel; 2 Infectious Diseases and Infection Control Units, Hillel Yaffe Medical Centre, Hadera, Israel; 3 Biochemistry Laboratory, Hillel Yaffe Medical Centre, Hadera, Israel; 4 Information Technology Department, Hillel Yaffe Medical Centre, Hadera, Israel; 5 Microbiology Laboratory, Hillel Yaffe Medical Centre, Hadera, Israel; 6 Faculty of Health Sciences, Ben-Gurion University of the Negev, Be’er Sheva, Israel; 7 Pharmacy Department, Hillel Yaffe Medical Centre, Hadera, Israel; 8 Clinical Laboratory Division, Hillel Yaffe Medical Centre, Hadera, Israel

## Abstract

**Objectives:**

To evaluate urinalysis (UA) performance for identifying single-uropathogen bacteriuria, characterize positive cultures after normal UA, compare laboratory and point-of-care platforms, and explore the resource implications of conditional culture.

**Methods:**

We retrospectively analyzed nonduplicate urine-culture episodes linked to same-day UA at a tertiary hospital in 2024. Pathological UA was defined as positive leukocyte esterase or nitrite. Diagnostic performance was assessed against single-uropathogen growth versus culture-negative reports. Selected culture-positive episodes underwent chart review. An exploratory model estimated resource use under a symptom- and UA-guided culture pathway.

**Results:**

Among 9,036 eligible culture episodes from 8,214 patients, 4,273 (47.3%) had same-day UA. The primary cohort comprised 3,969 episodes, including 925 single-uropathogen cases and 3,044 culture-negative controls. Pathological UA had 92.8% sensitivity (95% confidence interval, 91.1%–94.4%), 51.5% specificity (49.7%–53.2%), 36.8% positive predictive value (34.8%–38.7%), and 95.9% negative predictive value (94.9%–96.9%). Of 92 reviewed positive cultures after normal UA, 77 (83.7%) were classified as asymptomatic bacteriuria; seven of the remaining 15 episodes were adjudicated as probable urinary tract infection. The base-case model projected direct laboratory costs of ＄31,900 versus ＄102,700, with substantial variation across eligibility assumptions.

**Conclusions:**

Most positive cultures after normal UA represented asymptomatic bacteriuria, while culture-positive probable urinary tract infection was rare. These findings support a symptom-informed, UA-guided strategy with clinical overrides.

## Introduction

Urine cultures are frequently obtained in acute-care settings, including from patients without localizing urinary symptoms. Because bacteriuria is common and does not by itself establish urinary tract infection (UTI), positive cultures may prompt unnecessary antimicrobial treatment of asymptomatic bacteriuria (ASB), exposing patients to adverse effects and contributing to antimicrobial resistance.^(^ ^1^ ^,^ ^2^ ^)^ Diagnostic stewardship therefore seeks to improve both the clinical decision to obtain a urine culture and the laboratory pathway that follows.^(^ ^1^ ^,^ ^3^ ^,^ ^4^ ^)^

Conditional or reflex-to-culture pathways use urinalysis (UA) as a screening step, with culture performed only when predefined UA abnormalities are present. Leukocyte esterase (LE), nitrite, and microscopic pyuria have shown high negative predictive values (NPVs) for bacteriuria in selected populations, commonly ranging from 91% to 99%.^(^ ^5^ ^,^ ^6^ ^)^ However, predictive performance depends on the study population, prevalence of bacteriuria, specimen type, culture definition, and selected UA threshold.^(^ ^7^ ^,^ ^8^ ^)^ ASB may occur with or without pyuria.^(^ ^9^ ^)^ For conditional culture strategies, a key question is how often positive cultures following normal UA represent ASB rather than symptomatic UTI.

We evaluated the diagnostic performance of same-day UA for identifying single-uropathogen bacteriuria. Secondary objectives were to characterize positive cultures after normal UA, compare laboratory and point-of-care testing, and estimate the potential effects of conditional culture on culture volume and direct laboratory costs.

## Methods

### Study design and setting

We conducted a retrospective diagnostic-stewardship study at Hillel Yaffe Medical Center, a tertiary hospital in Israel, including all eligible urine-culture episodes submitted in 2024. Urine-culture processing was not conditional on UA findings. That year, the hospital recorded 139,530 emergency department visits and 153,406 inpatient-days. The institutional review board approved the study (0150-25-HYMC) and waived informed consent.

### Cohort assembly and data collection

Urine-culture records were linked to UA results recorded on the same calendar day. Extracted variables included patient age and sex, sampling unit, urine source, culture identification, semiquantitative growth, number of recovered organisms, laboratory comments, and UA results. Repeat cultures from the same patient within 72 hours were excluded, as were cultures from obstetrics, gynecology, pediatrics, and outpatient services. Specimens obtained from nephrostomies, ileal conduits, ureterostomies, other urinary diversions, or upper-tract drainage devices were also excluded.

### Microbiological classification and diagnostic endpoints

**Figure 1.**
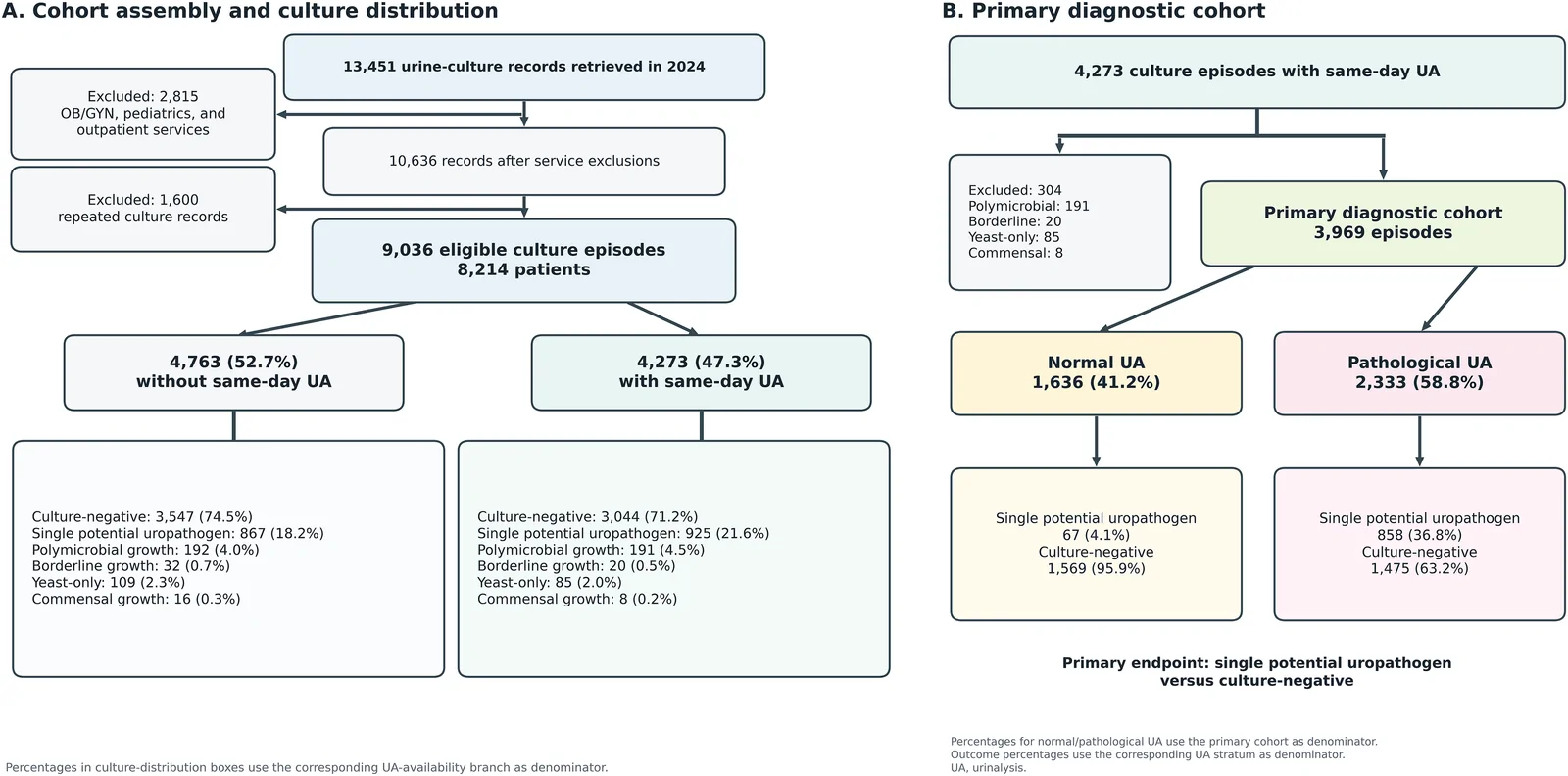
Figure 1 long description. Cohort assembly, culture classifications, same-day urinalysis availability, and the primary diagnostic cohort. Panel A shows the derivation of 9,036 eligible nonduplicate culture episodes from 13,451 retrieved records and the distribution of microbiological categories. Panel B shows the 4,273 episodes with same-day UA, the 304 exclusions from the primary end point, and the 3,969-case primary cohort stratified by normal versus pathological UA and culture outcome. UA, urinalysis.

The primary end point compared cultures growing a single potential uropathogen with culture-negative reports, defined as no growth or nonsignificant mixed growth without an identified uropathogen. Cultures classified as commensal, polymicrobial, borderline, or yeast-only were excluded (Figure 1). Classification incorporated organism identity and laboratory findings; neither a low colony count nor a “clinical correlation” comment alone determined exclusion. Polymicrobial cultures included distinct isolates of the same species and growth with incompletely identified additional organisms. This end point represents bacteriuria, not symptomatic UTI.

An exploratory ASB-informed end point reclassified chart-reviewed single-uropathogen cultures adjudicated as ASB as negative, irrespective of treatment. Partial and asymmetric chart review made this analysis susceptible to verification and classification bias. A broader supplementary analysis included single-uropathogen, polymicrobial, and borderline cultures as positive, retained culture-negative reports as negative, and excluded commensals and yeast-only cultures.

### Urinalysis acquisition and definitions

Point-of-care (POC) UA was performed using a Urisys® 1100 analyzer (Roche Diagnostics), mainly in the emergency department. Laboratory UA was performed in the biochemistry laboratory using a Cobas® 6500 analyzer (Roche Diagnostics), primarily for inpatient samples. Both pathways used Combur® 10 Test UX strips (Roche).

LE was reported as negative, 25, 100, or 500 cells/µL, and nitrite as negative or positive. Pathological UA was prespecified as any detectable LE (>0 cells/µL) or positive nitrite; blood on dipstick was not considered in defining a positive UA. When both laboratory and POC results were recorded on the same day, the overall analysis classified the case as pathological if either modality was pathological. Platform-specific analyses were performed separately, and direct platform comparisons were restricted to culture episodes with results from both modalities.

### Clinical record review

One investigator (RC), who was aware of the UA findings, reviewed culture-positive episodes with normal UA. Episodes were categorized as probable UTI, untreated ASB, treated ASB, or unresolved on the basis of documented urinary symptoms, systemic presentation, alternative diagnoses, imaging, microbiology, and treatment course. For comparison, consecutive eligible culture-positive episodes with pathological UA were reviewed until a similar sample size was reached; this comparison sample was neither randomized nor individually matched. Clinical review was used to characterize discordant episodes and to construct the exploratory ASB-informed end point, not as a uniformly applied clinical reference standard.

### Exploratory resource model

We modeled a two-stage conditional pathway: clinical eligibility determined whether UA was performed, and pathological UA determined whether culture followed. Base-case eligibility was 36%, based on the non-ASB fraction among reviewed positive cultures (67/188, 35.6%). Because this selected sample could not estimate eligibility among all requests, sensitivity analyses examined 20%–60% eligibility. We assumed 65% pathological UA among eligible episodes and a 70:30 laboratory-to-POC UA mix. Unit costs were ＄0.53 for laboratory UA, ＄2.61 for POC UA, ＄1.15 for culture inoculation, and ＄33.61 for identification and antimicrobial susceptibility testing (AST). Operational estimates were 2,600 identification and AST procedures under current practice and 765 in the base case. The model included only direct laboratory costs, excluding staff time, equipment depreciation, downstream antimicrobial use, and clinical consequences of non-actionable positive cultures.

### Statistical analysis

Categorical and continuous variables were summarized as counts (%) and medians (interquartile ranges [IQRs]), respectively, and compared using χ^2^ or Mann–Whitney U tests. Diagnostic performance was calculated from case-level 2 × 2 tables, with percentile 95% confidence intervals (CIs) from 2,000 patient-cluster bootstrap samples. A sensitivity analysis retained only the first eligible paired case for each patient, selected after applying the exclusions for the corresponding end point. Analyses used available observations without imputation. Exploratory analyses evaluated diagnostic performance by recorded culture collection setting (emergency department vs inpatient wards) and compared the sensitivity of LE, nitrite, and their combination between Gram-negative and Gram-positive single-organism cultures.

For the primary end point, platform-specific receiver operating characteristic (ROC) curves evaluated semiquantitative LE, binary nitrite, and a logistic model combining categorical LE with nitrite. Combined-model areas under the curve (AUCs) were considered apparent, exploratory estimates because fitting and evaluation used the same cohort. AUCs had 95% DeLong CIs; platform comparisons used paired DeLong tests in episodes with both measurements. Tests were two-sided, with *P* < .05 considered statistically significant. Analyses were performed in R version 4.4.0 (*tidyverse* and *pROC* packages). OpenAI ChatGPT assisted with code refinement and graphical formatting; the authors verified all analyses and plotted values.

## Results

### Cohort characteristics

Of 13,451 retrieved culture episodes, 9,036 nonduplicate eligible episodes from 8,214 patients remained (Figure 1). Median patient age was 70 years (IQR, 53–81), and 3,725 patients (45.3%) were female. Same-day UA was available for 4,273 culture episodes (47.3%); 2,612 (61.1%) were pathological and 1,661 (38.9%) were normal. Patients with at least one UA-associated case were older than those without UA (median, 72 vs 68 yr) and more often female (49.4% vs 41.4%; both *P* < .001). UA availability varied by unit (omnibus χ^2^ *P* < .001), from 5.7% in urology and 10.1% in intensive care to more than 60% in two internal medicine wards (Table 1).

**Table 1.** Patient characteristics and culture episodes according to same-day urinalysis availability Table 1 long description.

Characteristic	Total	UA available	UA unavailable	P value
**Panel A - Patient-level characteristics**				
Unique patients, n	8,214	4,069	4,145	—
Age, median (IQR), years	70 (53–81)	72 (56–82)	68 (51–79)	<0.001
Female sex, n (%)	3,725 (45.3)	2,010 (49.4)	1,715 (41.4)	<0.001
**Panel B - Culture episodes by unit**				
Emergency department cultures, n (%)	1,818 (20.1)	841 (46.3)	977 (53.7)	
Internal medicine 1	1,941 (21.5)	1,265 (65.2)	676 (34.8)	
Internal medicine 2	1,243 (13.8)	765 (61.5)	478 (38.5)	
Internal medicine 3	964 (10.7)	431 (44.7)	533 (55.3)	
Internal medicine 4	983 (10.9)	579 (58.9)	404 (41.1)	
Urology	1,158 (12.8)	66 (5.7)	1,092 (94.3)	
Intensive care unit	247 (2.7)	25 (10.1)	222 (89.9)	
Cardiac intensive care	130 (1.4)	48 (36.9)	82 (63.1)	
Other units	552 (6.1)	253 (45.8)	299 (54.2)	

IQR, interquartile range; UA, urinalysis. In Panel B, percentages in the Total column are based on all 9,036 culture episodes; percentages in the UA available and unavailable columns are calculated within each unit. The omnibus comparison of UA availability across units was significant (χ^2^ *P* < 0.001).

### Culture results

Among eligible episodes, 2,445 (27.1%) yielded an organism: 1,792 (19.8% of all episodes) grew a single potential uropathogen, 383 (4.2%) were polymicrobial, 52 (0.6%) were classified as borderline, 194 (2.1%) yielded yeast only, and 24 (0.3%) yielded commensal organisms. Among single-uropathogen cultures, *Escherichia coli* accounted for 49.1%, followed by *Klebsiella pneumoniae* (16.9%), *Proteus mirabilis* (6.8%), and *Pseudomonas aeruginosa* (6.2%) (Table 2).

**Table 2.** Distribution of organisms among 1,792 cultures with a single potential uropathogen Table 2 long description.

Organism	n	%
*Escherichia coli*	880	49.1
*Klebsiella pneumoniae*	302	16.9
*Proteus mirabilis*	122	6.8
*Pseudomonas aeruginosa*	112	6.2
*Enterococcus spp., not identified to species*	67	3.7
*Enterococcus faecalis*	54	3.0
*Citrobacter koseri*	48	2.7
*Streptococcus agalactiae*	39	2.2
*Klebsiella aerogenes*	30	1.7
*Enterobacter cloacae complex*	27	1.5
*Staphylococcus aureus*	18	1.0
*Providencia stuartii*	13	0.7
*Klebsiella oxytoca*	13	0.7
*Morganella morganii*	11	0.6
*Enterococcus faecium*	8	0.4
*Staphylococcus saprophyticus*	5	0.3
*Klebsiella spp.*	4	0.2
*Klebsiella variicola*	4	0.2
*Acinetobacter baumannii*	4	0.2
*Raoultella ornithinolytica*	4	0.2
Other organisms	27	1.5
**Total**	**1,792**	**100.0**

### Primary diagnostic performance

Among the 4,273 episodes with same-day UA, 304 were excluded from the primary analysis: 8 commensal, 191 polymicrobial, 20 borderline, and 85 yeast cultures. The primary cohort therefore comprised 3,969 episodes, including 925 single-uropathogen cases and 3,044 culture-negative controls. Pathological UA identified 858 cases and missed 67; among the 3,044 culture-negative controls, 1,475 had pathological UA and 1,569 had normal UA. Sensitivity was 92.8% (95% CI, 91.1%–94.4%), specificity 51.5% (49.7%–53.2%), positive predictive value (PPV) 36.8% (34.8%–38.7%), NPV 95.9% (94.9%–96.9%), positive likelihood ratio 1.91 (1.84–1.99), and negative likelihood ratio 0.14 (0.11–0.17) (Table 3).

**Table 3.** Diagnostic performance of pathological urinalysis for the primary microbiological and exploratory ASB-informed endpoints Table 3 long description.

End point	n	TP/FN	FP/TN	Sensitivity, % (95% CI)	Specificity, % (95% CI)	PPV, % (95% CI)	NPV, % (95% CI)	LR− (95% CI)
Primary: single potential uropathogen	3,969	858/67	1,475/1,569	92.8 (91.1–94.4)	51.5 (49.7–53.2)	36.8 (34.8–38.7)	95.9 (94.9–96.9)	0.14 (0.11–0.17)
Exploratory ASB-informed	3,969	816/14	1,517/1,622	98.3 (97.4–99.1)	51.7 (49.9–53.5)	35.0 (33.1–36.9)	99.1 (98.7–99.6)	0.03 (0.02–0.05)

ASB, asymptomatic bacteriuria; CI, confidence interval; FN, false negative; FP, false positive; LR−, negative likelihood ratio; NPV, negative predictive value; PPV, positive predictive value; TN, true negative; TP, true positive. The ASB-informed end point reclassified 95 chart-reviewed single-uropathogen ASB episodes as negative and is exploratory because clinical verification was partial and asymmetric.

Restriction to the first eligible paired case per patient yielded 3,802 episodes and produced similar sensitivity (92.6%), specificity (52.0%), PPV (37.2%), and NPV (95.8%).

In an exploratory analysis stratified by recorded culture collection setting, UA sensitivity was 96.3% in emergency department episodes and 91.7% in inpatient episodes, with corresponding specificities of 41.8% and 53.9%. NPVs were 96.8% and 95.7%, respectively (Supplementary Table 1).

### Clinical review and ASB-informed analysis

Records were reviewed for 92 culture-positive episodes with normal UA and 96 consecutively selected culture-positive episodes with pathological UA. ASB was identified in 77 of 92 normal-UA episodes (83.7%), including 6 treated cases, compared with 44 of 96 pathological-UA episodes (45.8%), including 11 treated cases. The selected normal-UA group had higher odds of ASB (odds ratio, 6.07; 95% CI, 2.93–12.9; *P* < .001). Because review was selective, these proportions do not estimate ASB prevalence in the full cohort.

Among the 15 normal-UA culture-positive episodes that were not classified as ASB, seven were adjudicated as probable UTI and eight remained unresolved. Fourteen grew a single potential uropathogen and were included among the 67 culture-positive episodes with normal UA in the primary analysis; the remaining case was polymicrobial and excluded from the primary analysis. Six of the seven probable UTI episodes had POC UA (Supplementary Table 2).

Within the primary cohort, 95 chart-reviewed single-uropathogen ASB episodes were reclassified as negative for the exploratory ASB-informed end point. This yielded 830 cases and 3,139 controls. Pathological UA identified 816 cases and missed 14, giving sensitivity of 98.3% (95% CI, 97.4%–99.1%), specificity of 51.7% (49.9%–53.5%), PPV of 35.0% (33.1%–36.9%), NPV of 99.1% (98.7%–99.6%), and a negative likelihood ratio of 0.03 (0.02–0.05) (Table 3). These estimates are exploratory because clinical verification was partial and asymmetric.

### Platform-specific ROC analyses

**Figure 2.**
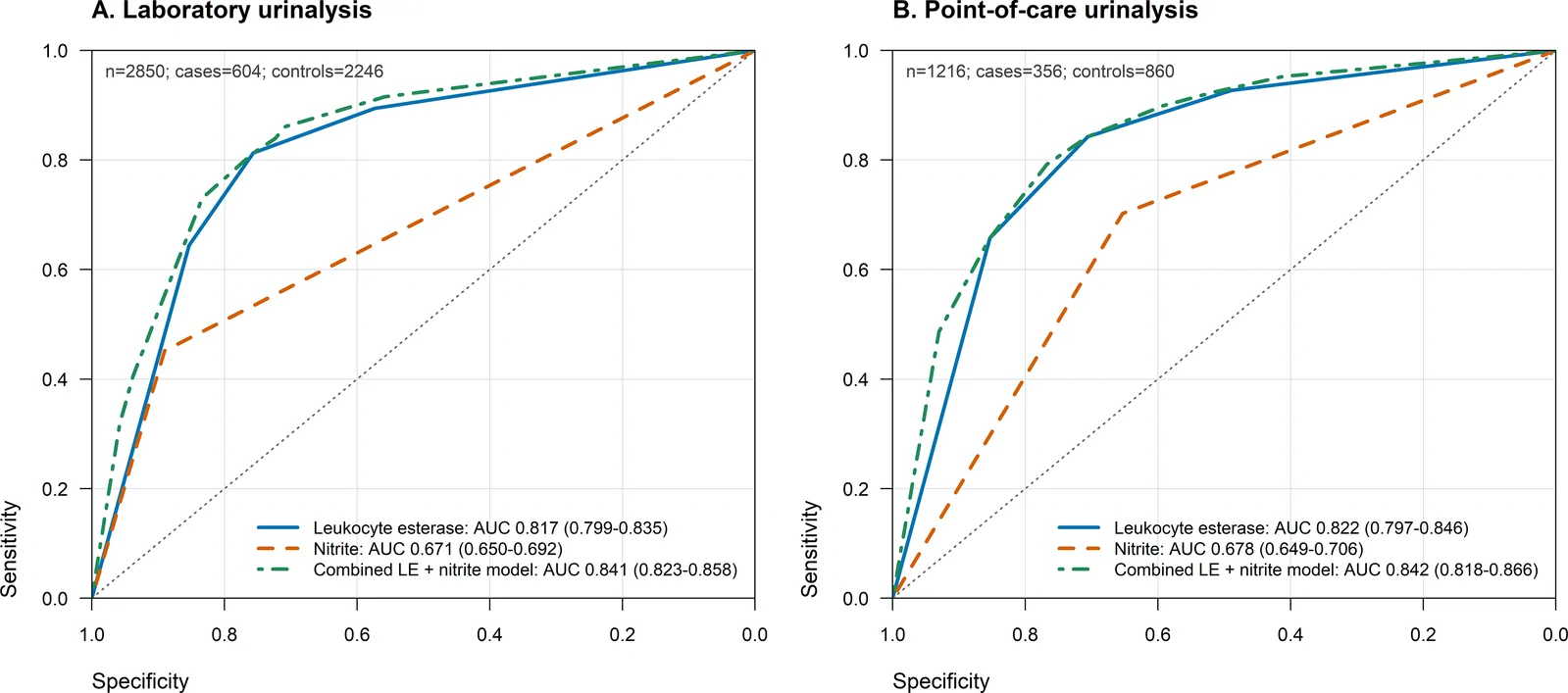
Figure 2 long description. Receiver operating characteristic curves for leukocyte esterase, nitrite, and the apparent leukocyte-esterase–plus-nitrite logistic model for the primary microbiological end point. (A) Laboratory-UA cohort (n = 2,850; 604 cases and 2,246 controls). (B) Point-of-care UA cohort (n = 1,216; 356 cases and 860 controls). AUCs were 0.817, 0.671, and 0.841, respectively, in the laboratory cohort and 0.822, 0.678, and 0.842 in the point-of-care cohort. The combined model was fitted and evaluated in the same cohort and should therefore be interpreted as an apparent, exploratory discrimination estimate. AUC, area under the receiver operating characteristic curve; LE, leukocyte esterase; POC, point of care; UA, urinalysis.

For the primary end point, the laboratory-UA cohort included 2,850 episodes (604 cases and 2,246 controls). AUCs were 0.817 (95% CI, 0.799–0.835) for LE, 0.671 (0.650-0.692) for nitrite, and 0.841 (0.823–0.858) for the apparent combined LE-plus-nitrite model. The POC cohort included 1,216 episodes (356 cases and 860 controls), with corresponding AUCs of 0.822 (0.797–0.846), 0.678 (0.649–0.706), and 0.842 (0.818–0.866), respectively (Figure 2).

Paired comparisons were available for 97 episodes (35 cases and 62 controls). For LE, the laboratory and POC AUCs were 0.839 and 0.773, respectively (paired DeLong *P* = .106). For nitrite, the corresponding AUCs were 0.731 and 0.678 (*P* = .364). Thus, the paired analysis did not demonstrate a statistically significant platform difference (Supplementary Figure 1).

In a broad sensitivity analysis that also classified polymicrobial and borderline cultures as positive (n = 4,180), sensitivity and specificity were similar to those in the primary analysis (Supplementary Table 3).

### Performance by organism Gram group

Among 925 single-organism bacterial cultures with same-day UA, 846 were Gram-negative and 79 were Gram-positive. Sensitivity of LE or nitrite positivity was 94.3% (95% CI, 92.7%–95.8%) for Gram-negative organisms and 75.9% (65.8%–84.8%) for Gram-positive organisms. Corresponding sensitivities were 92.3% versus 73.4% for LE and 57.2% versus 29.1% for nitrite (Supplementary Table 4).

### Exploratory resource model

Using rounded unit costs, current direct laboratory expenditure was approximately ＄102,700. In the illustrative 36% clinical-eligibility scenario, 3,253 episodes would undergo UA, 2,114 would proceed to culture, and approximately 765 isolates would undergo identification and susceptibility testing. Projected expenditure was approximately ＄31,900, a 68.9% reduction. Estimated savings ranged from 48.2% when 60% of requests were clinically eligible to 82.7% when 20% were eligible. These are modeled estimates rather than observed intervention effects.

## Discussion

This study identified a substantial opportunity to improve urine-culture use in our hospital. Same-day UA accompanied less than half of eligible culture episodes, and use varied markedly among wards. Moreover, 1,661 cultures were obtained and processed despite normal LE and nitrite, showing that even when UA was available it was not consistently used as a triage test. A similar disconnection between clinical indication, UA, and culture ordering has been described in acute-care settings^(10, 11)^. These findings support a two-stage stewardship pathway: clinicians should first establish an appropriate clinical indication for urine testing, and culture should then proceed primarily when UA is pathological. The observed ward variation also provides a practical basis for targeted education; our infection-control team subsequently initiated ward-level sessions emphasizing symptoms and review of UA results, although the effect of that intervention was not evaluated here.

For the primary microbiological end point, pathological UA had 92.8% sensitivity and a 95.9% NPV. A normal UA therefore did not exclude all single-uropathogen bacteriuria. The clinical interpretation, however, changed substantially after chart review. If UA alone had gated culture in the paired cohort, 1,661 of 4,273 cultures (38.9%) would not have proceeded. Among those 1,661 normal-UA episodes, 92 cultures were positive; 77 were classified as ASB, seven as probable UTI, and eight remained unresolved. Probable UTI therefore represented 0.4% of normal-UA episodes and 0.16% of all paired episodes. Even if all unresolved cases were conservatively treated as potential infections, the proportion among normal-UA episodes remained below 1%. Consistently, the exploratory ASB-informed end point yielded 98.3% sensitivity and a 99.1% NPV. Thus, the relevant finding is not that normal UA excludes all bacteriuria but that clinically meaningful infection after normal UA was uncommon in this cohort.

This interpretation is consistent with contemporary evidence. Advani *et al* found that the absence of trace LE or pyuria had an NPV of at least 95% for UTI and estimated that combined UA criteria could avoid nearly half of unnecessary cultures.^(^ ^5^ ^)^ More importantly, implementation studies have shown that conditional or reflex culture strategies can reduce urine cultures substantially. Munigala *et al* reported a 45% reduction after changing urine-testing orderables;^(^ ^12^ ^)^ a controlled six-hospital study reported a 21% reduction without an observed increase in Gram-negative bloodstream infection;^(^ ^13^ ^)^ and a recent ten-hospital implementation reported an 85% reduction, less antimicrobial use, and no detected increase in subsequent urinary-pathogen bloodstream infection or mortality.^(^ ^14^ ^)^ Together with the low absolute number of probable UTIs in our normal-UA group, these data provide reasonable reassurance for monitored implementation of a conditional pathway with a prespecified clinical override.

LE was more discriminative than nitrite, in agreement with prior studies,^(^ ^5^ ^,^ ^6^ ^)^ while the fitted LE-plus-nitrite model provided only a modest apparent improvement. In a pediatric study, Suresh et al likewise found higher sensitivity for LE than nitrite, particularly in young infants (92% vs 38%), illustrating the limitations of nitrite alone as a screening criterion for urine culture.^(^ ^15^ ^)^

Laboratory and POC UA showed similar overall discrimination. Although the laboratory LE AUC was numerically higher in the 97 paired episodes, the difference was not statistically significant; our data therefore do not support a claim of laboratory-platform superiority. Likewise, the observation that six of seven probable UTIs had POC UA is based on a very small selected group and should prompt platform-specific monitoring, not a conclusion that POC testing is less accurate. In our cohort, UA sensitivity was lower for Gram-positive than Gram-negative bacteriuria, with differences observed for both LE and nitrite (Supplementary Table 4). Semeniuk and Church likewise reported lower overall dipstick sensitivity for Gram-positive bacteriuria.^(^ ^16^ ^)^ Performance also varied by recorded culture collection setting, although NPVs were similar. These differences may reflect both patient mix and testing platform, whose contributions could not be separated in this analysis.

UA findings alone cannot distinguish symptomatic UTI from ASB, because bacteriuria must be interpreted in relation to urinary symptoms and clinical context^(^ ^8^ ^,^ ^9^ ^)^. However, a normal UA can still lower the probability of bacteriuria. A systematic review and meta-analysis in asymptomatic pregnant women found that a dipstick negative for both LE and nitrite reduced the estimated posttest probability of ASB from 8.0% to 2.6%. Although onsite tests showed high specificity, their sensitivity was insufficient to replace urine-culture screening in pregnancy, where missed ASB may have important clinical consequences.^(^ ^17^ ^)^ In our selected chart review, 83.7% of positive cultures after normal UA were classified as ASB, compared with 45.8% after pathological UA. Thus, a normal UA cannot determine whether an individual positive culture represents UTI or ASB, but in our reviewed sample, positive cultures following normal UA were more often classified as ASB than those following pathological UA.

The resource model illustrates the potential operational impact of a conditional testing pathway. Under an illustrative scenario in which 36% of episodes were clinically eligible for urine testing and culture was performed only after a pathological UA, projected annual direct laboratory costs decreased from approximately ＄102,700 to ＄31,900, a 68.9% reduction. In comparison, applying the UA gate alone to the observed paired cohort would have avoided 38.9% of cultures. The modeled cost savings additionally assumed that clinical eligibility would restrict initial urine testing. These projections are scenario-based rather than expected savings; across the sensitivity analyses, the estimated reduction ranged from 48.2% to 82.7%. The model also excluded staff time and downstream costs of unnecessary antimicrobial treatment. Previous urine-culture stewardship interventions reported laboratory cost savings^(^ ^12^ ^)^ and reduced antimicrobial use^(^ ^14^ ^)^, while emergency department costing studies further demonstrate the financial burden of unnecessary urine testing.^(^ ^18^ ^)^

## Limitations

This study has several limitations. Its retrospective design depended on the completeness of electronic documentation and may have misclassified symptoms or clinical context. UA availability was 47.3% overall and varied by ward, creating selection and spectrum bias. Same-day linkage did not ensure that UA and culture came from the same specimen or were obtained in a fixed sequence relative to antimicrobial exposure. Repeated episodes were retained, although patient-cluster bootstrap CIs and a first-case sensitivity analysis addressed within patient dependence.

Clinical adjudication was performed by one unblinded reviewer in selected culture-positive episodes, no uniform clinical reference standard was applied, and eight normal-UA episodes remained unresolved. The ASB-informed end point and the comparison of ASB by UA status are therefore susceptible to partial-verification and classification bias. These limitations make the exact clinical false-negative rate uncertain, although even counting all unresolved episodes as potential infections kept the absolute proportion below 1% among normal-UA episodes. Finally, the resource analysis was modeled rather than observed, depended on the assumed proportion of clinically eligible requests, and included direct laboratory costs only. Real-world implementation should measure overrides, delayed diagnoses, antimicrobial use, bloodstream infection, and other patient outcomes.

This study supports a symptom-informed conditional urine-culture pathway in which clinically eligible patients undergo UA and culture proceeds primarily after a pathological result, with an explicit override for strong clinical suspicion or prespecified high-risk situations. Although normal UA did not exclude every positive culture, probable UTI after normal UA was rare. This low absolute risk, together with evidence from implementation studies, provides reassurance that a monitored conditional strategy can substantially reduce unnecessary cultures, antimicrobial exposure, and laboratory costs without materially compromising detection of clinically significant infection.

## Supporting information

10.1017/ash.2026.10906.sm001 Cohen et al. supplementary material Cohen et al. supplementary material

## Data Availability

Available from the corresponding author upon reasonable request.

## Supplementary material

The supplementary material for this article can be found at https://doi.org/10.1017/ash.2026.10906

## Figure 1. Long description

Panel A: A flowchart showing the assembly of a cohort from 13,451 urine-culture records. Exclusions include records from OB/GYN, pediatrics, outpatient services, and repeated culture records, resulting in 9,036 eligible culture episodes from 8,214 patients. These episodes are divided into those with and without same-day urinalysis (UA), showing the distribution of microbiological categories. Panel B: A flowchart detailing the 4,273 culture episodes with same-day UA. Exclusions include polymicrobial, yeast-only, and commensal growths, resulting in a primary diagnostic cohort of 3,969 episodes. This cohort is stratified by normal and pathological UA and further categorized by culture outcomes, including single potential uropathogen and culture-negative results.

Navigate back to Figure 1..

## Table 1. Long description

A table comparing patient characteristics and culture episodes by urinalysis availability. The table has two panels: Panel A and Panel B. Panel A focuses on patient-level characteristics, while Panel B details culture episodes by unit. Panel A includes data on unique patients, age, and sex, with columns for total, UA available, and UA unavailable. Panel B lists culture episodes by different units, including emergency department, internal medicine, urology, intensive care, cardiac intensive care, and other units, with percentages for UA available and unavailable. Each row provides specific values for each characteristic or unit.

Navigate back to Table 1..

## Table 2. Long description

A table showing the distribution of organisms among 1,792 cultures with a single potential uropathogen. The table has 21 rows and 3 columns. The columns are labeled Organism, n, and %. Row 1: Escherichia coli, 880, 49.1 percent. Row 2: Klebsiella pneumoniae, 302, 16.9 percent. Row 3: Proteus mirabilis, 122, 6.8 percent. Row 4: Pseudomonas aeruginosa, 112, 6.2 percent. Row 5: Enterococcus spp., not identified to species, 67, 3.7 percent. Row 6: Enterococcus faecalis, 54, 3.0 percent. Row 7: Citrobacter koseri, 48, 2.7 percent. Row 8: Streptococcus agalactiae, 39, 2.2 percent. Row 9: Klebsiella aerogenes, 30, 1.7 percent. Row 10: Enterobacter cloacae complex, 27, 1.5 percent. Row 11: Staphylococcus aureus, 18, 1.0 percent. Row 12: Providencia stuartii, 13, 0.7 percent. Row 13: Klebsiella oxytoca, 13, 0.7 percent. Row 14: Morganella morganii, 11, 0.6 percent. Row 15: Enterococcus faecium, 8, 0.4 percent. Row 16: Staphylococcus saprophyticus, 5, 0.3 percent. Row 17: Klebsiella spp., 4, 0.2 percent. Row 18: Klebsiella variicola, 4, 0.2 percent. Row 19: Acinetobacter baumannii, 4, 0.2 percent. Row 20: Raoultella ornithinolytica, 4, 0.2 percent. Row 21: Other organisms, 27, 1.5 percent. Total: 1,792, 100.0 percent.

Navigate back to Table 2..

## Table 3. Long description

The table has two rows and nine columns. The columns are labeled End point, n, TP/FN, FP/TN, Sensitivity, % (95% CI), Specificity, % (95% CI), PPV, % (95% CI), NPV, % (95% CI), and LR- (95% CI). The row labels are Primary: single potential uropathogen and Exploratory ASB-informed. For the Primary endpoint, n is 3,969, TP/FN is 858/67, FP/TN is 1,475/1,569, Sensitivity is 92.8% (95% CI, 91.1%-94.4%), Specificity is 51.5% (49.7%-53.2%), PPV is 36.8% (34.8%-38.7%), NPV is 95.9% (94.9%-96.9%), and LR- is 0.14 (0.11-0.17). For the Exploratory ASB-informed endpoint, n is 3,969, TP/FN is 816/14, FP/TN is 1,517/1,622, Sensitivity is 98.3% (95% CI, 97.4%-99.1%), Specificity is 51.7% (49.9%-53.5%), PPV is 35.0% (33.1%-36.9%), NPV is 99.1% (98.7%-99.6%), and LR- is 0.03 (0.02-0.05).

Navigate back to Table 3..

## Figure 2. Long description

Panel A: Receiver operating characteristic curves for leukocyte esterase, nitrite, and the combined leukocyte-esterase plus nitrite model in the laboratory urinalysis cohort. The x-axis represents specificity, and the y-axis represents sensitivity. The blue line represents leukocyte esterase with an area under the curve (AUC) of 0.817. The orange dashed line represents nitrite with an AUC of 0.671. The green dashed line represents the combined model with an AUC of 0.841. Panel B: Receiver operating characteristic curves for the same models in the point-of-care urinalysis cohort. The blue line represents leukocyte esterase with an AUC of 0.822. The orange dashed line represents nitrite with an AUC of 0.678. The green dashed line represents the combined model with an AUC of 0.842.

Navigate back to Figure 2..
