# Supplementary material for: Feasibility of conditional urine culture based on pathological urinalysis: a retrospective diagnostic stewardship study
Source: Antimicrob Steward Healthc Epidemiol. 2026 Oct 16;6(1):e340. doi: 10.1017/ash.2026.10906 (PMC13760299; doi:10.1017/ash.2026.10906)
Supplement: Supplementary file 1 [file S2732494X26109061sup001.docx]

**Supplementary Table 1: Diagnostic performance of pathological urinalysis by recorded urine-culture collection setting**

| **Measure** | **Emergency department** | **Inpatient wards** |
| --- | --- | --- |
| Culture episodes, n | 801 | 3,168 |
| Single-uropathogen bacteriuria, n (%) | 215 (26.8) | 710 (22.4) |
| Culture-negative episodes, n | 586 | 2,458 |
| True positives, n | 207 | 651 |
| False negatives, n | 8 | 59 |
| False positives, n | 341 | 1,134 |
| True negatives, n | 245 | 1,324 |
| Sensitivity, % (95% CI) | 96.3 (93.4–98.7) | 91.7 (89.7–93.7) |
| Specificity, % (95% CI) | 41.8 (37.6–46.0) | 53.9 (51.8–55.7) |
| PPV, % (95% CI) | 37.8 (33.7–41.8) | 36.5 (34.2–38.7) |
| NPV, % (95% CI) | 96.8 (94.5–98.9) | 95.7 (94.7–96.8) |

Pathological urinalysis (UA) was defined as positive leukocyte esterase or nitrite; when both platforms were available, either positive result classified the episode as pathological. The primary endpoint compared single-uropathogen bacteriuria with culture-negative reports, excluding commensal, polymicrobial, borderline, and yeast-only cultures. Setting was assigned using the recorded culture ward, irrespective of UA platform. Percentile confidence intervals were calculated using 2,000 patient-cluster bootstrap replicates.

CI, confidence interval; NPV, negative predictive value; PPV, positive predictive value.

**Supplementary Table 2. Clinical characteristics of 15 chart-reviewed culture-positive episodes with normal urinalysis that were not classified as asymptomatic bacteriuria**

| **No.** | **Age/sex** | **Unit** | **UA platform** | **Culture** | **Key clinical features** | **Adjudication** | **UTI treatment** |
| --- | --- | --- | --- | --- | --- | --- | --- |
| 1 | 88/F | Internal medicine | Laboratory | *Enterococcus spp.* | Acute COVID-19; dementia; alternative respiratory infection; urinary symptoms could not be assessed | Unresolved | Yes |
| 2 | 65/F | Cardiac ICU | Laboratory | *K. pneumoniae* | Dementia; polymicrobial bloodstream infection; dysuria documented | Unresolved | Yes |
| 3 | 49/F | Internal medicine | POC | *E. coli* | Flank pain, urgency, vomiting, and fever; CT compatible with pyelonephritis | Probable UTI | Yes |
| 4 | 37/M | Emergency department | POC | *E. coli* | Acute scrotal pain without lower urinary symptoms | Unresolved | Yes |
| 5 | 48/F | Internal medicine | Laboratory | *E. coli* | Bladder cancer; intermittent self-catheterization; constipation and vomiting; urinary symptoms could not be assessed | Unresolved | No |
| 6 | 69/F | Internal medicine | Laboratory | *K. pneumoniae* | Acute kidney injury requiring dialysis; same organism in blood and urine | Probable UTI | Yes |
| 7 | 59/M | Internal medicine | Laboratory | *K. pneumoniae* | Acute coronary syndrome; dysuria; bacteriuria was not treated | Unresolved | No |
| 8 | 67/F | Emergency department | POC | ESBL *E. coli* | Intellectual disability; febrile convulsions; no clear alternative source; urinary symptoms could not be assessed | Unresolved | Yes |
| 9 | 80/F | Internal medicine | POC | *E. coli* | Fever, weakness, and lower urinary symptoms | Probable UTI | Yes |
| 10 | 28/F | Emergency department | POC | *E. coli* | Abdominal pain; ureteral dilation and periureteral stranding on CT | Probable UTI | Yes |
| 11 | 76/M | Internal medicine | POC | *E. faecalis* | Acute kidney injury; ureteral stone and hydronephrosis requiring urgent intervention | Probable UTI | Yes |
| 12 | 23/F | Internal medicine | POC | ESBL *K. pneumoniae* | Lower urinary symptoms and flank tenderness; afebrile | Probable UTI | Yes |
| 13 | 88/F | Internal medicine | Laboratory | *Enterococcus spp.* | Dementia, urinary retention, and cough; urinary symptoms could not be assessed | Unresolved | No |
| 14 | 31/F | Internal medicine | POC | *K. pneumoniae* | Lower urinary symptoms; same organism previously; nitrite positive two days later | Probable UTI | Yes |
| 15 | 77/F | Internal medicine | POC | *E. coli + K. pneumoniae* | Septic shock; pressure-injury infection considered the more likely source; urinary symptoms could not be assessed | Unresolved | Yes |

All 15 episodes had negative leukocyte esterase and nitrite. Fourteen grew a single potential uropathogen and were included among the 67 primary false-negative cultures; case 15 was polymicrobial and was excluded from the primary microbiological endpoint. CT, computed tomography; ESBL, extended-spectrum beta-lactamase; ICU, intensive care unit; POC, point of care; UA, urinalysis; UTI, urinary tract infection.

**Supplementary Table 3. Diagnostic performance of pathological urinalysis for the broad microbiological endpoint**

| **Endpoint** | **n** | **TP/FN** | **FP/TN** | **Sensitivity, % (95% CI)** | **Specificity, % (95% CI)** | **PPV, % (95% CI)** | **NPV, % (95% CI)** | **LR− (95% CI)** |
| --- | --- | --- | --- | --- | --- | --- | --- | --- |
| Broad microbiological endpoint | 4,180 | 1,055/81 | 1,475/1,569 | 92.9 (91.4–94.3) | 51.5 (49.8–53.3) | 41.7 (39.8–43.6) | 95.1 (94.0–96.1) | 0.14 (0.11–0.17) |

The broad endpoint classified cultures growing a single potential uropathogen, polymicrobial growth, or borderline growth as positive; culture-negative reports were controls, and commensal and yeast-only cultures were excluded. CI, confidence interval; FN, false negative; FP, false positive; LR−, negative likelihood ratio; NPV, negative predictive value; PPV, positive predictive value; TN, true negative; TP, true positive.

**Supplementary Table 4. Sensitivity of urinalysis criteria by organism Gram classification among single-organism bacterial cultures**

| **UA criterion** | **Gram-negative (n=846)** | | **Gram-positive (n=79)** | |
| --- | --- | --- | --- | --- |
|  | **Positive/total** | **Sensitivity, % (95% CI)** | **Positive/total** | **Sensitivity, % (95% CI)** |
| LE positive | 781/846 | 92.3 (90.5–94.1) | 58/79 | 73.4 (63.3–82.3) |
| Nitrite positive | 484/846 | 57.2 (54.0–60.6) | 23/79 | 29.1 (19.0–39.2) |
| LE or nitrite positive | 798/846 | 94.3 (92.7–95.8) | 60/79 | 75.9 (65.8–84.8) |

Analysis included 925 single-organism bacterial culture episodes with same-day UA. Organisms were grouped by Gram classification based on their recorded identity. When both UA platforms were available, positivity on either platform counted as positive for the corresponding criterion. Percentile 95% CIs were calculated using 2,000 patient-cluster bootstrap replicates. Specificity and predictive values were not estimated because this analysis included only culture-positive episodes.

CI, confidence interval; LE, leukocyte esterase; UA, urinalysis.

**Supplementary Figure 1 Legend**

Paired comparison of laboratory and point-of-care urinalysis in the 97 culture episodes with results from both platforms (35 single-uropathogen bacteriuria cases and 62 culture-negative controls). (A) Receiver operating characteristic curves for leukocyte esterase: laboratory AUC 0.839 and point-of-care AUC 0.773 (paired DeLong p=0.106). (B) Receiver operating characteristic curves for nitrite: laboratory AUC 0.731 and point-of-care AUC 0.678 (p=0.364). AUC, area under the receiver operating characteristic curve; LE, leukocyte esterase; POC, point of care; UA, urinalysis.
